# Trematode genetic patterns at host individual and population scales provide insights about infection mechanisms

**DOI:** 10.1017/S0031182023000987

**Published:** 2023-11

**Authors:** Simão Correia, Sergio Fernández-Boo, Luísa Magalhães, Xavier de Montaudouin, Guillemine Daffe, Robert Poulin, Manuel Vera

**Affiliations:** 1 Department of Biology, CESAM, University of Aveiro, 3810-193 Aveiro, Portugal; 2 Aquatic and Animal Health Group, CIIMAR, University of Porto, 4450-208 Matosinhos, Portugal; 3 Department of Zoology, Genetics and Physical Anthropology, Campus Terra, University of Santiago de Compostela, 27002 Lugo, Spain; 4 Department of Zoology, University of Otago, 9054 Dunedin, New Zealand; 5 CNRS, UMR EPOC, Station Marine, University of Bordeaux, F-33120 Arcachon, France; 6 Université de Bordeaux, CNRS, Observatoire Aquitain des Sciences de l'Univers, F-33615 Pessac, France

**Keywords:** *Bucephalus minimus*, *Cerastoderma edule*, clonal diversity, COI, host–parasite interactions, parasite, population genetics

## Abstract

Multiple parasites can infect a single host, creating a dynamic environment where each parasite must compete over host resources. Such interactions can cause greater harm to the host than single infections and can also have negative consequences for the parasites themselves. In their first intermediate hosts, trematodes multiply asexually and can eventually reach up to 20% of the host's biomass. In most species, it is unclear whether this biomass results from a single infection or co-infection by 2 or more infective stages (miracidia), the latter being more likely *a priori* in areas where prevalence of infection is high. Using as model system the trematode *Bucephalus minimus* and its first intermediate host cockles, we examined the genetic diversity of the cytochrome c oxidase subunit I region in *B. minimus* from 3 distinct geographical areas and performed a phylogeographic study of *B. minimus* populations along the Northeast Atlantic coast. Within localities, the high genetic variability found across trematodes infecting different individual cockles, compared to the absence of variability within the same host, suggests that infections could be generally originating from a single miracidium. On a large spatial scale, we uncovered significant population structure of *B. minimus*, specifically between the north and south of Bay of Biscay. Although other explanations are possible, we suggest this pattern may be driven by the population structure of the final host.

## Graphical abstract

**Figure:**
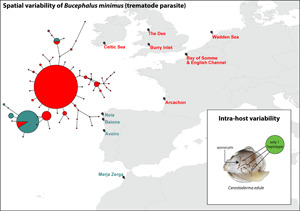

## Introduction

With about 45 000 species described in a wide range of ecosystems, trematodes are one of the most common and widespread group of parasites (Carlson *et al*., 2020). They can be found at almost across all trophic levels of dynamic food chains (Bartoli and Gibson, 2007). Trematodes are an important component of ecosystem biodiversity with significant impacts at the host individuals (SchulteOehlmann *et al*., 1997; Curtis *et al*., 2000; Thieltges, 2004), host populations (Fredensborg *et al*., 2005) and ecosystem communities (Poulin, 1999; Mouritsen and Poulin, 2002; Goedknegt *et al*., 2016). Besides, by contributing to the nutrient cycle, acting as indicators of environmental changes or as proxy of environmental diversity (due to multi-host life cycles), trematode presence may indicate a healthy and resilient ecosystem (Johnson *et al*., 2010; Hatcher and Dunn, 2011).

Trematodes have a complex life cycle that alternates between free-living and parasitic stages. The miracidium, the trematode larva hatched from an egg, infects the first intermediate host (usually a mollusc) and transforms into sporocysts or rediae (parasitic stage). At this stage, sporocysts or rediae, through asexual multiplication, produce cercariae (free-living stage) that emerge from the first host to infect the second intermediate host (a vertebrate or invertebrate) where they settle as metacercariae. In the trematode's final host (a vertebrate), after ingestion of the second host, metacercariae develop into adult flukes, reproduce sexually, and complete the life cycle (Cribb *et al*., 2003; Bartoli and Gibson, 2007). In the first intermediate host, sporocyst stages are overtly destructive, replacing host tissue and reaching up to 20% of the host's biomass (Dubois *et al*., 2009; Preston *et al*., 2013), with direct consequences for host reproduction (Carballal *et al*., 2001), growth (Bowers, 1969) and energy demand (Jokela *et al*., 1993), leading to eventual host death (Thieltges, 2006). In most species, it is currently unknown whether this sporocyst biomass results from a single miracidium, that excludes other miracidia by predation or intraspecific competition, or from co-infection. If co-infection is the rule, trematode invasion may result in a burden that the host might not be able to bear (Fredensborg and Poulin, 2005; Mideo, 2009). On the other hand, co-infection can, occasionally, benefit the host by lessening the overall burden of infection, by reducing parasite infection success, or by strengthening the host's immune response, leading to higher resistance to infection (Dumont *et al*., 2007; Balmer *et al*., 2009).

Determining the genetic diversity of trematode sporocysts within and among individual first intermediate hosts is therefore important to understand the biology, behaviour and evolutionary patterns of these parasites. Nonetheless, current knowledge regarding biology of these parasites, and particularly about the sporocyst life stage, is still very scarce, despite trematodes' wide distribution and importance to the ecosystem. Few studies have focused on the conspecific diversity of trematode sporocysts within the first intermediate host (Rauch *et al*., 2005; Keeney *et al*., 2007; Lagrue *et al*., 2007), showing that the likelihood of infection by conspecifics increased with the prevalence of the parasite in the community (Keeney *et al*., 2008; Louhi *et al*., 2013). Moreover, at the population level, the host has a significant impact on genetic diversity and population structure of parasites, with substantial gene flow occurring in parasite species with efficient dispersion mechanisms (Agola *et al*., 2009; Feis *et al*., 2015). However, most studies of trematode genetic diversity have focused on intermediate and final hosts, with particular emphasis on trematodes with harmful impacts on human (Theron *et al*., 2004; Bell *et al*., 2006; Balmer *et al*., 2009) or socio-economically important species, namely fish (Vilas *et al*., 2003; Criscione and Blouin, 2006).

*Bucephalus minimus* is a marine trematode parasite that occurs in several aquatic systems along the Northeast Atlantic coast and Mediterranean Sea (Magalhães *et al*., 2015). In the Atlantic area, this parasite infects the European edible cockle, *Cerastoderma edule*, which serves as the first intermediate host when a miracidium penetrates its tissue. In cockles, the prevalence of this parasite varies greatly among coastal systems and season; depending on the time since infection, the parasite's dry mass in infected cockles can range from 1 to 20% of the total living tissue within the cockle shell (Magalhães *et al*., 2015; de Montaudouin *et al*., 2021). *Bucephalus minimus* initially infects the cockle's gonad and digestive gland but promptly spreads to other parts of the host, eventually invading the entire body (Desclaux *et al*., 2002; de Montaudouin *et al*., 2009). Infection by *B. minimus* results in castration (Carballal *et al*., 2001) and energy consumption (Dubois *et al*., 2009), leading to starvation and autolysis of the host's digestive tract. This parasite is considered as one of the most harmful trematode parasites infecting *C. edule* (Magalhães *et al*., 2015; de Montaudouin *et al*., 2021). Inside cockles, *B. minimus* produces sporocysts and cercariae through asexual multiplication, which emerge and infect the goby *Pomatoschistus* spp. (second intermediate host), where they encyst and develop into metacercariae. The final host, *Dicentrarchus labrax*, the European seabass, is infected after consumption of parasitized gobies. Metacercariae develop into adult flukes and produce eggs, through sexual reproduction, to complete the cycle (Pina *et al*., 2009; Magalhães *et al*., 2015).

Due to the limited information regarding this trematode's sporocyst stages, the primary goal of the present study was to assess the genetic variability of the cytochrome c oxidase subunit I (COI) region of *B. minimus* sporocyst DNA within and among the first intermediate host, *C. edule*. We tested the hypothesis that higher genetic variability at the host individual scale (resulting in 2 or more haplotypes among sporocysts in a single cockle) are more common in localities with high prevalence of infection, where joint infections should be more frequent by chance alone. As our second goal, a phylogeographic study of *B. minimus* was also carried out combining information available in the literature and from samples taken on cockle beds that were examined in this study for the first time (i.e. Iberian Peninsula and Great Britain).

## Materials and methods

### *Bucephalus minimus* samples

**Figure 1.**
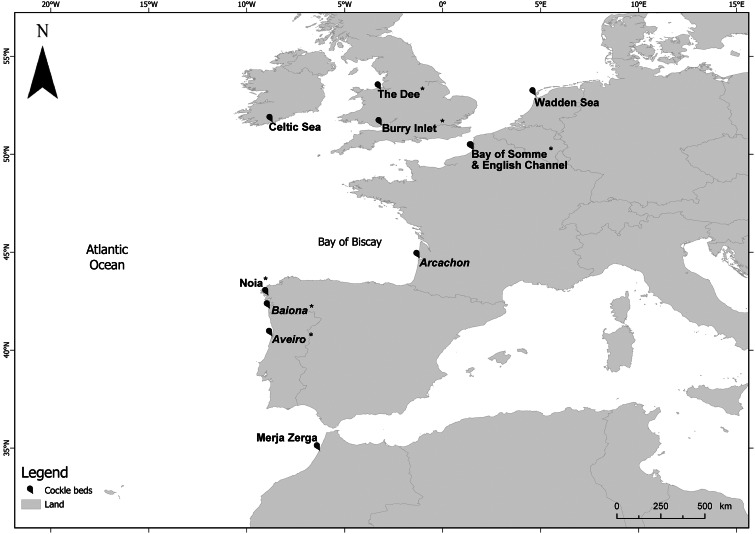
Geographical location of the *Cerastoderma edule* cockle beds sampled for the study of *Bucephalus minimus* genetic variability at host level (Aveiro, Baiona and Arcachon [in italics]) and for phylogeographic analysis. *Cockle beds sampled for the first time in this study.

The genetic variability of *B. minimus* sporocyst haplotypes within the same host was studied by collecting specimens present in the first intermediate host, the edible cockle, at 3 different beds with different prevalence along the European Atlantic coast: Ria de Aveiro, Aveiro, Portugal (lowest prevalence [Magalhães *et al*., 2018]); de la Ramallosa Lagoon, Baiona, Spain (moderate prevalence [Intecmar, 2021]); and Île aux Oiseaux, Arcachon, France (with high levels of prevalence [Magalhães *et al*., 2015]) (Fig. 1). Adult cockles (between 20 and 30 mm shell length) were haphazardly collected at low tide and dissected in the laboratory to morphologically identify *B. minimus* infection. The flesh was then transferred and observed under a stereomicroscope by carefully compressing it between 2 sterilized glass slides. Four sporocyst replicates were extracted per cockle (in a total of 5 cockles per sampling site) using forceps, and preserved separately in 100% ethanol at −20°C. All material was sterilized between samples. To enhance the possibility of different clones, sporocysts were taken from different infected tissues of the same cockle (i.e. 1 from the foot, 1 from the gills and 2 from the digestive gland).

### DNA isolation, amplification and sequencing

Genomic DNA extraction from *B. minimus* specimens was performed using E.Z.N.A Mollusc DNA kit (Omega Bio-Tek, Norcross, GA, USA) in accordance with the manufacturer's instructions. Nanodrop was used to assess DNA concentration, and, if needed, aliquots were created to dilute DNA to approximately 30 ng μL^−1^.

The mitochondrial COI fragment was amplified using the MplatCOX1-dF (5′-TTW CIT TRG ATC ATA AG-3′) and MplatCOX1-dR (5′-TGA AAY AAY AII GGA TCI CCA CC-3′) primers (Moszczynska *et al*., 2009), resulting in sequences of 587 bp. The polymerase chain reaction (PCR) was carried out in a final volume of 20 μL composed of 1X reaction buffer, 2.5 mm MgCl_2_, 100 μm deoxynucleotide triphosphates (dNTPs), 0.5 μm of forward and reverse primers, 0.65 units of ThermoFisher AmpliTaq Gold DNA polymerase (ThermoFisher, Waltham, MA, USA) and 60 ng of DNA. The PCR programme employed had an initial denaturation step for 10 min at 95°C, followed by 35 cycles of 30 s at 94°C, 30 s at 50°C and 1 min at 72°C and a final extension of 10 min at 72°C. Following this initial PCR, a second PCR was carried out using the same conditions previously described but using 2 μL of PCR product instead of DNA. The amplified PCR products were analysed by electrophoresis through a 1% agarose gel dyed with SYBR safe DNA Gel Stain (Invitrogen, Carlsbad, CA, USA) and visualized under UV light.

PCR products were enzymatically purified with ExoSAP mix (10 μL of PCR product, 0.6 units of EXO I [DNA nuclease] and 0.3 units of shrimp alkaline phosphatase [SAP] for a final volume of 12 μL) under the following conditions: 60 min at 37°C and 15 min at 85°C. Purified PCR products were sequenced using the ABI Prism BigDye^TM^ Terminator v3.1 Cycle Sequencing Kit protocol on an ABI Prism 3730 xl automatic sequencer (Applied Biosystems, Foster City, CA, USA). All sequences obtained in this study were deposited in GenBank (accession numbers: OQ625925–OQ625936; Table 1). Variable sites were manually checked using the SEQSCAPE 2.5 program (Applied Biosystems) and aligned using ClustalW algorithm implemented on BioEdit v.7.2.5 (Hall, 1999) with LaB haplotype (GenBank accession number: KF880429.1) as reference. Identification of the different haplotypes in the *B. minimus* specimens analysed was carried out using the software DNAsp v5.10 (Librado and Rozas, 2009). Finally, to check for the presence of premature stop codons, COI haplotypes identified were translated to amino acid sequences using the flatworm mtDNA code in the online software EMBOSS Transeq (Rice *et al*., 2000; Goujon *et al*., 2010).

**Table 1.** Accession number for *B. minimus* COI gene DNA sequences downloaded (with reference) and deposited (in bold) in GenBank

Haplotype name	GenBank accession number	Reference
LaA	KF880428.1	Feis *et al*. (2015)
LaB	KF880429.1	Feis *et al*. (2015)
LaC	KF880430.1	Feis *et al*. (2015)
LaD	KF880431.1	Feis *et al*. (2015)
LaE	KF880432.1	Feis *et al*. (2015)
LaF	KF880433.1	Feis *et al*. (2015)
LaG	KF880434.1	Feis *et al*. (2015)
LaH	KF880435.1	Feis *et al*. (2015)
LaI	KF880436.1	Feis *et al*. (2015)
LaJ	KF880437.1	Feis *et al*. (2015)
LaK	KF880438.1	Feis *et al*. (2015)
LaL	KF880439.1	Feis *et al*. (2015)
LaM	KF880440.1	Feis *et al*. (2015)
LaN	KF880441.1	Feis *et al*. (2015)
LaO	KF880442.1	Feis *et al*. (2015)
LaP	KF880443.1	Feis *et al*. (2015)
LaQ	KF880444.1	Feis *et al*. (2015)
LaR	KF880445.1	Feis *et al*. (2015)
LaS	KF880446.1	Feis *et al*. (2015)
LaT	KF880447.1	Feis *et al*. (2015)
LaU	KF880448.1	Feis *et al*. (2015)
LaV	KF880449.1	Feis *et al*. (2015)
LaW	KF880450.1	Feis *et al*. (2015)
LaX	KF880451.1	Feis *et al*. (2015)
LaY	KF880452.1	Feis *et al*. (2015)
LaZ	KF880453.1	Feis *et al*. (2015)
LaAA	KF880454.1	Feis *et al*. (2015)
LaAB	KF880455.1	Feis *et al*. (2015)
LaAC	KF880456.1	Feis *et al*. (2015)
LaAD	KF880457.1	Feis *et al*. (2015)
LaAE	KF880458.1	Feis *et al*. (2015)
LaAF	KF880459.1	Feis *et al*. (2015)
LaAG	KF880460.1	Feis *et al*. (2015)
LaAH	KF880461.1	Feis *et al*. (2015)
LaAI	KF880462.1	Feis *et al*. (2015)
LaAJ	KF880463.1	Feis *et al*. (2015)
LaAK	KF880464.1	Feis *et al*. (2015)
LaAL	KF880465.1	Feis *et al*. (2015)
LaAM	KF880466.1	Feis *et al*. (2015)
LaAN	KF880467.1	Feis *et al*. (2015)
LaAO	KF880468.1	Feis *et al*. (2015)
LaAP	KF880469.1	Feis *et al*. (2015)
LaAQ	KF880470.1	Feis *et al*. (2015)
LaAR	KF880471.1	Feis *et al*. (2015)
LaAS	KF880472.1	Feis *et al*. (2015)
LaAT	KF880473.1	Feis *et al*. (2015)
LaAV	KF880474.1	Feis *et al*. (2015)
LaAW	KF880475.1	Feis *et al*. (2015)
LaAX	KF880476.1	Feis *et al*. (2015)
LaAY	KF880477.1	Feis *et al*. (2015)
LaAZ	KF880478.1	Feis *et al*. (2015)
LaBA	KF880479.1	Feis *et al*. (2015)
LaBB	KF880480.1	Feis *et al*. (2015)
LaBC	KF880481.1	Feis *et al*. (2015)
**BmA**	**OQ625925**	**This study**
**BmB**	**OQ625926**	**This study**
**BmC**	**OQ625927**	**This study**
**BmD**	**OQ625928**	**This study**
**BmE**	**OQ625929**	**This study**
**BmF**	**OQ625930**	**This study**
**BmG**	**OQ625931**	**This study**
**BmH**	**OQ625932**	**This study**
**BmI**	**OQ625933**	**This study**
**BmJ**	**OQ625934**	**This study**
**BmK**	**OQ625935**	**This study**
**BmL**	**OQ625936**	**This study**

### Data analysis

#### *Bucephalus minimus* genetic variability at host level

To determine the genetic variability of *B. minimus* specimens within the same cockle and among different cockles, haplotypes found in the different tissues of each analysed cockle were compared using BioEdit. A haplotype network was built using the obtained data, identifying haplotypes for each cockle with a different colour. The haplotype network was constructed by calculating the distance (based on number of base pair differences) between DNA sequences and determining the number of mutations between haplotypes using ‘pegas’ and ‘ape’ packages of R Statistical Software v.4.2.2 (Paradis, 2010; Paradis and Schliep, 2019).

#### Phylogeographic analysis

For phylogeographic analyses, together with the specimens collected in the present study, DNA extractions of 13 specimens from 7 cockle beds sampled as part of the COCKLES Interreg project (http://cockles-project.eu/) were sequenced as previously described. Samples were available for Aveiro, Portugal (1 sample), Noia, Spain (1 sample), Arcachon, France (1 sample), Bay of Somme, France (4 samples), The Dee, Wales (2 samples), Burry Inlet, Wales (2 samples) and Wadden Sea, the Netherlands (2 samples). Additionally, the analysis included another 54 COI sequences available in the GenBank database retrieved in January 2023 (see Feis *et al*., 2015 and Table 1). In total, sequences represented specimens from 11 different cockle beds located in 7 countries, covering a large part of the natural distributional range of cockles in the Atlantic area (Fig. 1).

Phylogenetic relationships were studied using different and complementary approaches. First, a haplotype network was computed for the full dataset as previously described, which included information regarding the cockle bed in which the haplotypes were found and the frequency of occurrence. Identification of unique haplotypes present in the dataset was carried out with DnaSP v.5.10 (Librado and Rozas, 2009). Using MEGA X software (Kumar *et al*., 2018), the Hasegawa–Kishino–Yano nucleotide substitution rate (HKY) with a *γ* value of 0.655 and invariable sites of 0.728 was identified as the most probable nucleotide substitution model for our data. Phylogenetic trees were constructed using exclusively the different haplotypes identified and the nucleotide substitution model described above. Maximum likelihood (ML) and neighbour-joining rooted and unrooted trees were constructed using the R Statistical Software v.4.2.2. The rooted tree was created using the COI sequences of *Rhipidocotyle* sp. (a trematode from the same family as *B. minimus*, Bucephalidae, GenBank accession number: KM538111.1) and *Himasthla quissetensis* (a trematode that infects cockles as second intermediate host but from a different family, Himasthlidae; GenBank accession number: MN272732.1) as outgroups. The sequences were trimmed to 540 bp to remove missing data. The unrooted trees were constructed using the full 587 bp sequences of *B. minimus*. The robustness of the branches for the phylogenetic trees was estimated with 1000 bootstrap replicates and a likelihood ratio test was performed based on the minimum Akaike information criterion values for ML. All phylogeographic analyses were performed with the ‘ape’, ‘pegas’, ‘ggtree’ and ‘phangorn’ packages of R Statistical Software v.4.2.2 (Paradis, 2010; Schliep, 2011; Yu *et al*., 2017; Paradis and Schliep, 2019).

Genetic diversity parameters, calculated as haplotype diversity (*h*) and nucleotide diversity (*π*), were estimated within each cockle bed studied using Arlequin v.3.5.1.3 (Excoffier *et al*., 2005). The HKY model is not available in this software. For this reason, the Tamura–Nei (TN) model with a *γ* value of 0.639 (similar to the HKY and identified as the third best option for MEGA X) was used for *π* estimations. Genetic structure and population differentiation were assessed with global and pairwise coefficients of population differentiation applying the TN with *γ* value of 0.639 substitution rate (ϕ_ST_ values) with Arlequin. Analysis of molecular variance (AMOVA) applying different models of *a priori* clustering (based on host population genetics [Souche *et al*., 2015; Vera *et al*., 2022] and observed data – see results) was carried out to study the distribution of genetic variation within (ϕ_SC_) and among (ϕ_CT_) bed groups using Arlequin. The significance for all the ϕ statistics was evaluated with 10 000 permutations.

## Results

### *Bucephalus minimus* genetic variability at host level

During this study, a total of 210 cockles were analysed, of which 17 were found to be infected with *B. minimus* (5 each in Aveiro and Baiona and 7 in Arcachon). The prevalence of *B. minimus* in the cockle beds sampled varied from 3.3% in Aveiro (Portugal) to 23.3% in Arcachon (France). In Baiona (Spain), *B. minimus* was present in 16.7% of the sampled cockles. Five infected cockles per bed were used to extract 4 sporocysts per cockle, yielding a total of 60 samples. Fifty-six samples were successfully sequenced, while 4 samples, from a single cockle from Arcachon (France), were not successfully sequenced due to DNA extraction problems. From the 56 sequenced sporocysts, belonging to 14 infected cockles, 12 different haplotypes were identified, with 5, 3 and 4 haplotypes found in Aveiro, Baiona and Arcachon, respectively (Table 2). Six of the identified haplotypes were characterized for the first time (named as BmA–BmF; see Table 1).

**Table 2.** Coordinates of each cockle bed, the number of analysed cockles (*N*_cockles_), number of parasites sequenced (*N_B. minimus_*), prevalence of *B. minimus*, number of haplotypes (*k*), number of polymorphic sites (PS) and haplotype composition (between parentheses the number of individuals bearing the same haplotype when different from one)

Cockle bed	Coordinates	*N*					
		Cockles	*B. minimus*	Prevalence	*k*	P*S*	Haplotype composition
Aveiro	40.710123, −8.704596	5	20	3.3%	5	8	BmA, BmB, BmC, BmD, BmE
Baiona	42.117020, −8.820283	5	20	16.7%	3	3	BmF, LaD, LaE (3)
Arcachon	44.690111, −1.182944	4	16	23.3%	4	5	BmG, LaAE, LaAQ, LaAW
**General**		**14**	**56**	**8.1%**	**12**	**14**	

**Figure 2.**
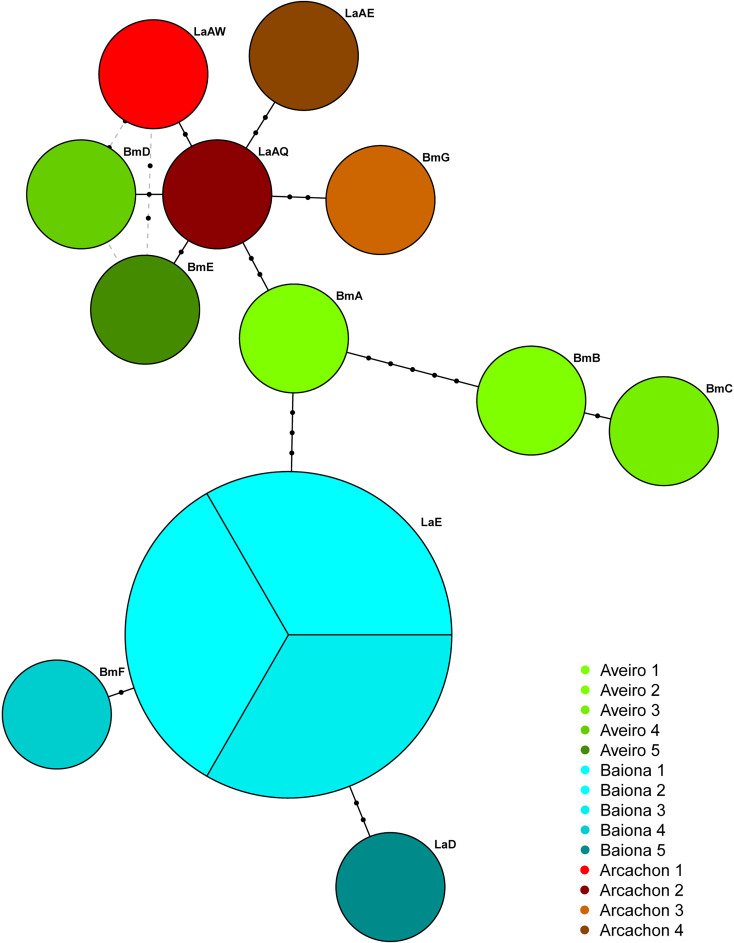
Haplotype network of *Bucephalus minimus* samples from Aveiro (Portugal), Baiona (Spain) and Arcachon (France) based on genetic distance (number of base pair differences) of cytochrome c oxidase subunit 1 (COI) gene sequences. Different haplotypes with respective names are represented by circles, with circle size proportional to observed frequency. Inferred mutation steps are shown by black dots. Colours depict samples taken from the same cockle.

All sporocysts of *B. minimus* from the same cockle had identical haplotype, however *B. minimus* haplotypes identified in different cockles from the same bed were different, except in Baiona where the haplotype found in 3 different cockles was identical (haplotype LaE; see Table 2 and Fig. 2). Moreover, haplotypes were not shared among the 3 beds (Fig. 2).

### Phylogeographic analysis

From the 69 DNA sequenced samples (56 for the *B. minimus* haplotype genetic variability at host level study, and 13 from different European sites selected from the COCKLES project), 12 resulted in novel haplotypes (Table 1). No premature STOP codons were identified in these sequences (data not shown). Thus, when GenBank resources were included, a total of 162 COI gene sequences of *B. minimus* specimens from 11 cockle beds were analysed. From these available sequences, 66 represented unique haplotype sequences. Shared haplotypes (i.e. those found in more than 1 cockle bed) accounted for 17% of the total. The LaAQ haplotype was the most prevalent and abundant haplotype, occurring 42 times across 6 different beds. On the other hand, 83% of the haplotypes were exclusively found in a single bed, with several reported only once (i.e. singletons). Arcachon presented the highest number of different *B. minimus* haplotypes detected (19, see Table 3).

**Table 3.** Haplotype frequency for *B. minimus* COI gene per cockle bed including haplotype diversity (*h* ± standard deviation) and nucleotide diversity (*π* ± standard deviation)

Haplotype	Merja Zerga	Aveiro	Baiona	Noia	Arcachon	Bay of Somme	English Channel	Celtic Sea	Burry Inlet	The Dee	Wadden Sea	Sum
LaA	0	0	0	0	0	0	0	0	0	0	1	1
LaB	20	0	0	0	2	0	0	0	0	0	0	22
LaC	0	0	0	0	1	0	0	0	0	0	0	1
LaD	0	0	1	0	1	0	0	0	0	0	0	2
LaE	0	0	3	0	2	0	1	0	0	0	0	6
LaF	0	0	0	0	0	0	0	1	0	0	0	1
LaG	0	0	0	0	0	0	1	0	0	0	0	1
LaH	0	0	0	0	0	0	0	0	0	0	1	1
LaI	5	0	0	0	0	0	0	0	0	0	0	5
LaJ	0	0	0	0	0	0	0	8	1	0	0	9
LaK	0	0	0	0	0	0	0	1	0	0	0	1
LaL	1	0	0	0	0	0	0	0	0	0	0	1
LaM	0	0	0	0	0	0	0	1	0	0	0	1
LaN	0	0	0	0	0	0	0	1	0	0	0	1
LaO	0	0	0	0	1	0	0	0	0	0	0	1
LaP	0	0	0	0	0	0	0	0	0	0	1	1
LaQ	0	0	0	0	0	0	0	0	0	0	1	1
LaR	0	0	0	0	0	0	1	1	0	1	1	4
LaS	0	0	0	0	0	0	0	1	0	0	0	1
LaT	0	0	0	0	0	0	0	1	0	0	0	1
LaU	0	0	0	0	0	0	0	0	0	0	1	1
LaV	0	0	0	0	0	0	0	2	0	0	0	2
LaW	0	0	0	0	1	0	0	0	0	0	0	1
LaX	0	0	0	0	0	0	1	0	0	0	0	1
LaY	0	0	0	0	0	0	0	1	0	0	0	1
LaZ	0	0	0	0	0	0	0	1	0	0	0	1
LaAA	0	0	0	0	1	0	0	0	0	0	0	1
LaAB	0	0	0	0	1	0	0	0	0	0	0	1
LaAC	0	0	0	0	1	0	0	0	0	0	0	1
LaAD	0	0	0	0	0	0	0	0	0	0	1	1
LaAE	0	0	0	0	2	0	0	0	0	0	0	2
LaAF	0	0	0	0	0	0	4	1	0	0	0	5
LaAG	0	0	0	0	0	0	0	1	0	0	0	1
LaAH	0	0	0	0	0	0	0	1	0	0	0	1
LaAI	0	0	0	0	0	0	1	0	0	0	0	1
LaAJ	0	0	0	0	0	0	0	0	0	0	1	1
LaAK	0	0	0	0	0	0	0	0	0	0	1	1
LaAL	0	0	0	0	0	0	1	0	0	0	1	2
LaAM	0	0	0	0	0	0	0	1	0	0	0	1
LaAN	0	0	0	0	1	0	2	0	0	0	0	3
LaAO	0	0	0	0	0	0	0	0	0	0	1	1
LaAP	0	0	0	0	0	0	0	0	0	0	1	1
LaAQ	0	0	0	0	7	2	12	4	1	0	16	42
LaAR	0	0	0	0	0	0	1	0	0	0	0	1
LaAS	0	0	0	0	1	0	0	0	0	0	0	1
LaAT	0	0	0	0	0	0	1	0	0	0	1	2
LaAV	0	0	0	0	0	0	0	0	0	0	1	1
LaAW	0	0	0	0	2	0	0	0	0	0	0	2
LaAX	0	0	0	0	0	0	1	0	0	0	0	1
LaAY	0	0	0	0	1	0	0	0	0	0	0	1
LaAZ	0	0	0	0	1	0	0	0	0	0	0	1
LaBA	0	0	0	0	0	0	1	0	0	0	0	1
LaBB	0	0	0	0	0	0	2	0	0	0	0	2
LaBC	0	0	0	0	1	0	0	0	0	0	0	1
BmA	0	1	0	0	0	0	0	0	0	0	0	1
BmB	0	1	0	0	0	0	0	0	0	0	0	1
BmC	0	1	0	1	0	0	0	0	0	0	0	2
BmD	0	1	0	0	0	0	0	0	0	0	0	1
BmE	0	1	0	0	0	0	0	0	0	0	0	1
BmF	0	0	1	0	0	0	0	0	0	0	0	1
BmG	0	0	0	0	1	0	0	0	0	0	0	1
BmH	0	0	0	0	0	1	0	0	0	0	0	1
BmI	0	0	0	0	0	1	0	0	0	0	0	1
BmJ	0	0	0	0	0	0	0	0	0	1	0	1
BmK	0	1	0	0	0	0	0	0	0	0	0	1
BmL	0	0	0	0	1	0	0	0	0	0	0	1
**Sum**	26	6	5	1	29	4	30	27	2	2	30	162
***h***	0.3846 ± 0.1017	1.0000 ± 0.0962	0.7000 ± 0.2184	1.0000 ± 0.0000	0.9384 ± 0.0340	0.8333 ± 0.2224	0.8299 ± 0.0632	0.9003 ± 0.0461	1.0000 ± 0.5000	1.0000 ± 0.5000	0.7241 ± 0.0922	
***Π***	0.000688 ± 0.000732	0.007572 ± 0.005013	0.002082 ± 0.001834	0.0000 ± 0.0000	0.006253 ± 0.003626	0.003468 ± 0.002899	0.003077 ± 0.002031	0.005543 ± 0003282	0.001719 ± 0.002426	0.005170 ± 0.005961	0.003172 ± 0.002079	

**Figure 3.**
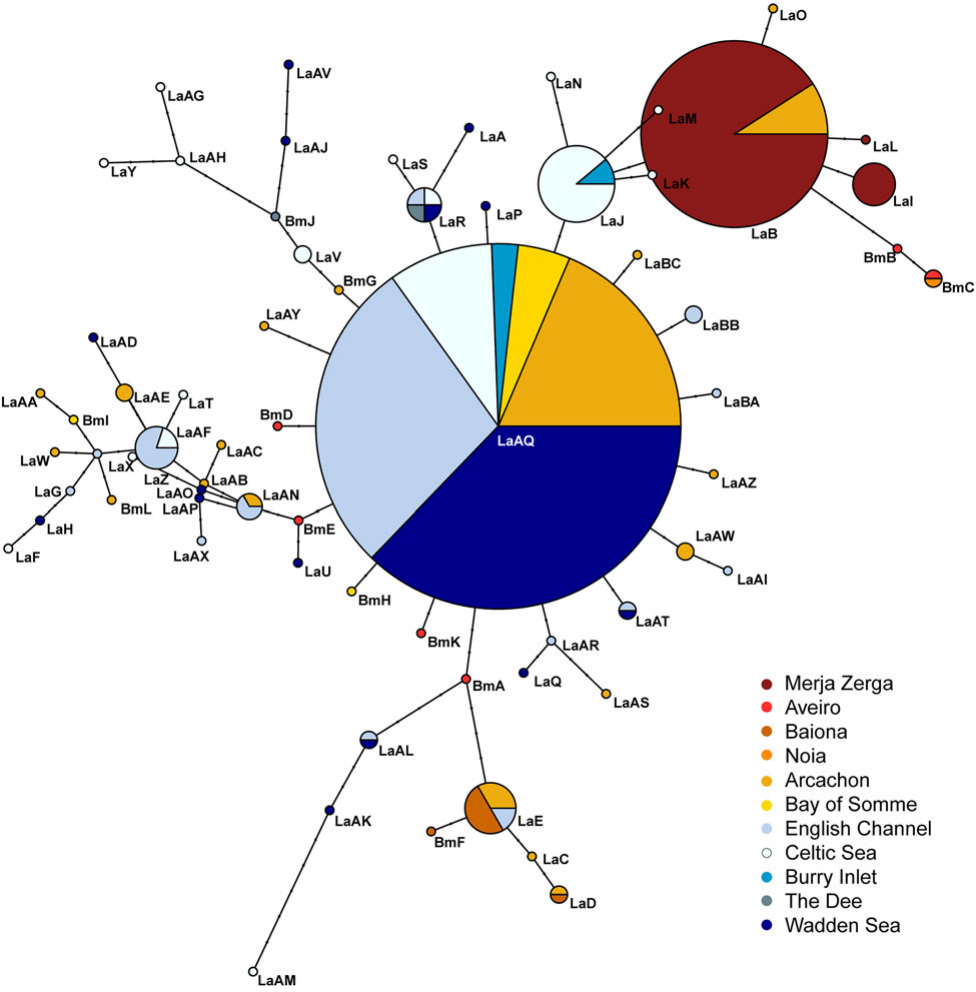
Haplotype network of *Bucephalus minimus* based on genetic distance (number of base pair differences) of cytochrome c oxidase subunit 1 (COI) gene sequences across 11 beds of *Cerastoderma edule* where *Bucephalus minimus* specimens were collected. Different haplotypes with respective names are represented by circles, with circle size proportional to observed frequency. Inferred mutation steps are shown by black dots. Colours depict sample location.

**Figure 4.**
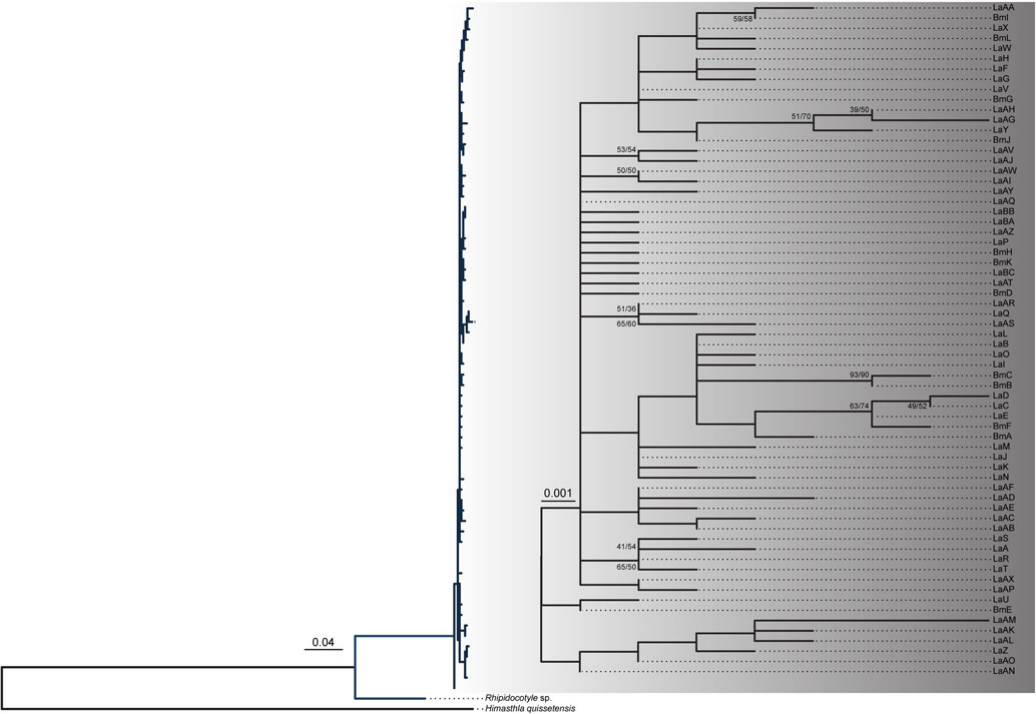
Maximum likelihood phylogenetic tree based on cytochrome c oxidase subunit I (COI) gene sequences of *Bucephalus minimus* haplotypes from this study and retrieved from GenBank database. *Rhipodocotyle* sp. and *Himasthla quissetensis* haplotypes were used as outgroups. Numbers at nodes represent the percentage of replicate trees in which the associated taxa clustered together in the bootstrap test (1000 replicates) using neighbour-joining and maximum likelihood methods (NJ/ML). The scale bar indicates the distances in substitutions per nucleotide.

Due to the high variability found in the COI region, the analysis of *B. minimus* haplotypes across the various cockle beds produced a complex network made up of several closely connected haplotypes and associated mutational steps, with no more than 5 mutations separating any 2 successive haplotypes identified. A common haplotype (LaAQ), observed in several beds situated north of Arcachon (44°N), was located in the centre of the network, from which numerous other haplotypes diverged in a star-like pattern. These haplotypes were exclusively found in a single bed or shared between relatively close beds (Fig. 3). Nonetheless, haplotype clusters (i.e. haplogroups) were identified in specific geographic areas, and beds from the South (Merja Zerga, Aveiro, Baiona and Noia) and North (Bay of Somme, English Channel, Celtic Sea, Burry Inlet, The Dee and Wadden Sea) did not share any haplotype, with the exception of the LaE haplotype identified in Baiona, Arcachon and the English Channel. Phylogenetic relationships observed in the network were also confirmed with the phylogenetic trees (Fig. 4).

The Arcachon bed, located in the centre of cockle's distributional range between the northern and southern geographic areas, exhibited the highest number of detected haplotypes (19), sharing haplotypes with locations from both regions (Table 3). Excluding Noia, where only 1 individual was analysed, haplotype diversity ranged from 0.3846 in Merja Zerga to 1.0000 in beds where all individuals analysed had a distinct haplotype (Aveiro, Burry Inlet and The Dee). Nucleotide diversity ranged from 0.0007 in Merja Zerga to 0.0076 in Baiona (Table 3). The high diversity and the haplotype distribution among locations were also reflected in the ϕ_ST_ values. Global ϕ_ST_ for the whole region was 0.2922 (*P* value < 0.001). Many pairwise ϕ_ST_ values resulted in significant differences, although many comparisons between close locations were non-significant, mainly among those involving northern beds (Supplementary Table S1). Moreover, all pairwise ϕ_ST_ values involving Merja Zerga were high and highly significant (*P* value < 0.001), suggesting the singularity of this bed (global ϕ_ST_ in the whole region excluding Merja Zerga = 0.1706, *P* value < 0.001). These results suggest the presence of one northern group (composed by Bay of Somme, English Channel, Celtic Sea, Burry Inlet, The Dee and Wadden Sea) more homogeneous genetically (ϕ_ST_ = 0.0295, *P* value = 0.073) than the southern one (composed by Merja Zerga, Aveiro, Baiona and Noia; ϕ_ST_ = 0.6694, *P* value < 0.001), with Arcachon representing a potential contact region between both geographic areas. The ϕ_ST_ value in the northern group increased up to 0.0336 (*P* value = 0.018) when Arcachon was included, while this value decreased in the southern group when this location was included although it remained quite high (ϕ_ST_ = 0.4184, *P* value < 0.001). Hence, these results suggest a closer relationship of Arcachon with the northern group. AMOVA analysis assigned 34.58% of the genetic differentiation to differences between northern and southern groups (ϕ_CT_ = 0.3458, *P* value = 0.008), this percentage being three times higher than those assigned to differences among beds within groups (ϕ_SC_ = 0.2109, *P* value < 0.001, percentage of genetic differentiation = 13.80%). The AMOVA model including Arcachon in the northern group yielded similar values (ϕ_CT_ = 0.3100, *P* value = 0.006, percentage of genetic differentiation = 31.00%; ϕ_SC_ = 0.1567, *P* value < 0.001, percentage of genetic differentiation = 10.81%). This model assigned a higher percentage of genetic differentiation among groups and a lower percentage to differences among beds within groups than the model including Arcachon in the southern group (ϕ_CT_ = 0.1327, *P* value = 0.048, percentage of genetic differentiation = 13.27%; ϕ_SC_ = 0.2299, *P* value < 0.001, percentage of genetic differentiation = 19.13%), suggesting a more coherent grouping of the beds in the former model.

## Discussion

### *Bucephalus minimus* genetic variability at host level

Co-infection by multiple parasites, from the same or different species, within the same host is a well-recognized phenomenon in the parasitological literature (Poulin, 2001; Read and Taylor, 2001). This pattern has been extensively studied for several parasite species, namely with an impact on human health (Theron *et al*., 2004; Bell *et al*., 2006). For example, in the case of malaria, more than 5 strains have been found to be infecting the same host (Bell *et al*., 2006). The same trend was observed for the trematode parasite *Schistosoma mansoni* within their second and final host (Theron *et al*., 2004). Similar to what is observed for metacercarial or adult stages of trematode parasites, it would be anticipated that different clones would infect the same first intermediate host when thousands of eggs per infected definitive host are shed into the water column, i.e. thousands of miracidia hatching within metres of each other. This was observed for some trematode species (Rauch *et al*., 2005; Keeney *et al*., 2007; Lagrue *et al*., 2007). In the present study, only 1 COI haplotype was found inside each infected cockle (regardless of the samples' origin), in contrast with what has been previously recorded. Nevertheless, it should be noted that in the present study only the COI region (maternally inherited) was sequenced, while for previous studies, microsatellite markers were used to identify individual variability. In fact, microsatellite markers are more accurate for population structure analysis and individual identification since they are highly variable polymorphic regions (Abdul-Muneer, 2014) and inherited from both parents. Despite its limitations, the high genetic variability of the COI region found in this species (12 haplotypes out of 14 analysed cockles), as well as in each of the studied beds individually (no repeated haplotypes in 2 out of 3 studied cockle beds – see Table 2), suggests that possibly only 1 individual (i.e. 1 miracidium) of *B. minimus* infects the host and/or prevails inside it. Unfortunately, no microsatellite markers are currently developed for this trematode species or other closely related species (which could have been used by cross-validation). Therefore, further studies using nuclear markers that are either highly polymorphic (such as microsatellites) or in a high number (single nucleotide polymorphisms, SNPs) – which would reduce the probability of random sharing of multilocus genotypes among individuals – will be necessary to confirm our results.

In the Ria de Aveiro, given the low prevalence of *B. minimus*, the presence of only 1 parasite haplotype per host was not surprising. There is a well-known upwelling mechanism offshore of this coastal lagoon (Queiroz *et al*., 2012), resulting in low water temperature and consequently lower prevalence and abundance of trematode parasites compared to other coastal systems where cockles are distributed (Correia *et al*., 2020). Trematodes are highly sensitive to temperature, both in their free-living and parasitic stages (Thieltges and Rick, 2006; Selbach and Poulin, 2020). For example, the production and hatching rate of trematode eggs are positively correlated with temperature, peaking under ideal thermal conditions (Morley, 2012; Morley and Lewis, 2017). The same happens with cercarial multiplication within and emergence from the first intermediate host (Poulin, 2006; de Montaudouin *et al*., 2016). Hence, Ria de Aveiro may have fewer free-living stages (miracidia) in the water and might take longer for the cycle to complete. Adding to it the high density of the host found in the area, a parasite intensity dilution effect (as occurs in other regions, e.g. Magalhães *et al*., 2017) might also contribute to a single conspecific parasite infection in each individual host, as we observed. However, the same pattern (i.e. 1 haplotype per host) was found for cockles from de la Ramallosa lagoon (Baiona) and Île aux Oiseaux (Arcachon), where the prevalence of *B. minimus* can exceed 20%. Double infection by trematode sporocysts in cockles is rare (Magalhães *et al*., 2015, 2020), most likely due to rare exposure of the host to a second miracidium. However, occurrence of co-clone infection has been shown to arise when the prevalence of hosts with trematode sporocyst infection rises (Keeney *et al*., 2008; Louhi *et al*., 2013). Therefore, the rationale above that relates temperature and low prevalence as causes of single haplotype infection lacks support in Baiona (Spain) and Arcachon (France) and suggests again that *B. minimus* infection may originate from a single miracidium.

Alternatively, the genetic diversity at host level may be determined by the infection mechanisms of these parasites, such as intraspecific competition. The presence of a single *B. minimus* haplotype per host could also be explained by the production of substances that could change host chemical attractiveness (Baiocchi *et al*., 2017) or be toxic against competitors (Burman, 1982; Selva *et al*., 2009). This is true for nematode parasites, but there is no information on trematodes. However, synthesis of harmful chemicals seems highly unlikely as it would impact the trematode's own clones. Nonetheless, to test these predictions, specific experiments would need to be conducted.

Another explanation may be that cockles with multiple infection (i.e. those with more than 1 haplotype) are rare because they incur higher mortality rates than single infections. This stage of the trematode life cycle is highly deleterious for the host and can lead to mass mortality events during outbreaks (Thieltges, 2006; de Montaudouin *et al*., 2021). Periods of high prevalence may also result in a rise in co-infections, which may heighten the host's susceptibility and mortality. Theoretical and empirical studies provide conflicting findings, and it is therefore unclear whether susceptibility and subsequent host death are directly connected to co-infection by conspecific parasites (Read and Taylor, 2001; Davies *et al*., 2002; Alizon and van Baalen, 2008). Regardless, a study conducted using different strains of the trematode *S. mansoni* in their host showed an increase in overall pathogenicity (Davies *et al*., 2002).

As an alternative and excluding the above scenarios, it is possible that after parasite settlement, co-infection becomes unlikely due to alterations in host characteristics. For instance, the nematode *Acanthocheilonema viteae* induces an immune response by the host to further infection by free-living larvae, with no effect on the nematode's persistence within the host (Rajakumar *et al*., 2006). The extensive use of host tissues, particularly the gonad and digestive gland (Dubois *et al*., 2009), could be another reason for the absence of co-infection. This exhaustive use of resources might prevent the settlement of other parasite individuals. Interestingly, co-infection with another trematode species at the same sporocyst stage is exceedingly rare and less frequent than expected (in terms of probability), without predominance by either species (Magalhães *et al*., 2020). The exhaustive use of tissue preventing the establishment of further miracidia seems to be the most likely explanation for our findings. Thus, the present results cannot fully demonstrate whether co-infection by conspecifics is present or not due to the limited sample size and the genetic marker used. They do, however, suggest that co-infections might be rare and not the rule, and that, for a co-infection to occur, different miracidia must infect simultaneously or within a short period of time. Further studies using codominant nuclear DNA molecular markers, such as microsatellites or SNPs (unavailable to date for *B. minimus*), or attempts at experimental infection of previously infected cockles, would help to determine whether co-infections are possible and confirm our results.

### *Bucephalus minimus* phylogeography

The COI sequences amplified in this study, as well as those available from the literature (Feis *et al*., 2015), were all grouped together, with short branches separating each sequence, indicating that the sequences all belong to the same species throughout the entire cockle distributional range, with no evidence of any subspecies.

The haplotype network constructed resulted in a complex pattern with several different haplotypes, generally with few mutational steps between them (at most 5 steps between 2 adjacent haplotypes) in accordance with the previous study by Feis *et al*. (2015). These results are typical of a population with relatively stable age structure and a fixed ratio of natural growth (i.e. stable demography) (Loewe and Hill, 2010). The absence of shared haplotypes among the various beds (most haplotypes were present in a single bed) supports this conclusion. These results were expected and consistent with previous reports on this species (Feis *et al*., 2015). Trematodes have a complex life cycle that involves a vertebrate, usually a fish or a bird, as definitive host (Cribb *et al*., 2003). In the case of *B. minimus*, its final host is the European seabass, which has limited migratory capacity compared to birds (de Pontual *et al*., 2023). This limited mobility results in a higher degree of isolation between populations of *B. minimus*.

In our study, 2 haplogroups were identified, one located south of the Bay of Biscay (including Merja Zerga, Aveiro, Baiona and Noia) and the other to the north (including Bay of Somme, English Channel, Celtic Sea, Burry Inlet, The Dee and Wadden Sea). These groups did not share haplotypes except one (LaE). However, this division also coincides with the cockle beds where a lower number of individuals were assessed (see Table 3). Besides, it is noteworthy that there is a core haplotype present in 6 different beds from which most of the other haplotypes diverge. This haplotype has only been identified in the northernmost beds. However, it may represent an ancestral haplotype that has spread across the different regions, and then undergone evolutionary mutations that are specific to each bed in which it occurs. For example, the haplotype network created for the *B. minimus* genetic variability at host level study (Fig. 2) revealed higher similarity of Aveiro (south group) and Arcachon (central/north group) haplotypes than Aveiro and Baiona (south group) haplotypes due to a parsimonious position (position 50 of our alignment) in the COI sequences where a cytosine replaces a thymine in all Baiona haplotypes. Consequently, the presence or absence of haplotypes in a particular bed should be interpreted cautiously, especially for southern beds.

Despite the limitations previously described, the population genetics of *B. minimus* exhibited similar geographic clusters as those of its first intermediate host, *C. edule* [structured as northwards and southwards of French Brittany (Vera *et al*., 2022)]. Cockle dispersal mainly occurs during their larval stages (Martel and Chia, 1991), when *B. minimus* cannot infect them (Magalhães *et al*., 2015). It may also occur through human-mediated movements, although no information is available regarding this possibility. Therefore, it is more logical to expect a correlation with the population genetic structure of seabass, the most mobile host in the trematode life cycle (Zemmer *et al*., 2020). Two distinct genetic population units, one in the Atlantic area and another in the Mediterranean, have been previously identified for seabass (Souche *et al*., 2015), with a slight genetic differentiation in the Atlantic area observed south of the Strait of Gibraltar (Morocco) attributed to a hybrid zone between 2 evolutionary lineages (Lemaire *et al*., 2005; Vandeputte *et al*., 2019). A genomic survey conducted with more than 2700 molecular markers (i.e. SNPs) throughout the Atlantic area revealed the presence of 3 different groups weakly differentiated and geographically distributed. Thus, all Atlantic wild fish belonged to a single group, except specimens from the northern North Sea (i.e. Norway) and the Strait of Gibraltar (AQUATRACE, 2017), matching the results of Souche *et al*. (2015). This structure (see Fig. S1) could explain the high *B. minimus* population differentiation found when Merja Zerga (located on the Moroccan coast) was compared with the remaining beds, although more beds south of the Strait of Gibraltar need to be studied, along with greater sample sizes from southern beds, especially in the Iberian Peninsula, to corroborate this explanation. Following seabass genetic structure, the other cockle beds analysed may be included within the same genetic group. Despite this homogeneity, Quéré *et al*. (2010) identified genetic differentiation between seabass from the Bay of Biscay and North Sea using a molecular marker (microsatellite) under selection associated to the somatolactin gene, although this pattern was not confirmed either by other candidate genes or adaptive variation screenings (Souche *et al*., 2015; AQUATRACE, 2017). Moreover, an electronic tagging study in seabass has identified the Bay of Biscay as a potential hybridization zone for several subpopulations of seabass from various Atlantic coast locations (de Pontual *et al*., 2023), which could account for the region's high haplotype diversity and differentiation in the population genetics of *B. minimus*. In any event, the effect of selective processes on seabass population structure cannot be ruled out and could explain the current genetic structure in *B. minimus*, with the Bay of Biscay (here represented by Arcachon bed) being a possible hybrid region between northern and southern groups. Thus, the genetic structure among *B. minimus* population uncovered in our study may reflect local adaptation of the parasite to the most common host genotypes occurring in each of the regions sampled (e.g. Sasal *et al*., 2000). One possible approach to further investigate this possibility would be to conduct co-phylogeographic analyses between the genetic structure of the parasite and that of their cockle and seabass hosts (e.g. Nieberding *et al*., 2004; Nieberding and Olivieri, 2007).

In conclusion, with a single COI haplotype observed per host, *B. minimus* genetic variability at the host level was very limited. This suggests that a single miracidium may be infecting *C. edule*, possibly indicating the existence of strong mechanisms operating in the background to reduce the likelihood of multiple infections. However, the processes involved are currently poorly understood, with a long way to go to fully comprehend trematode host–parasite interactions. Particularly, when discussing the early stage of the parasite's life cycle (sporocysts), laboratory studies are required to confirm the likelihood of co-infections and the defence mechanisms involved. Similar to the population structure of its first host, *C. edule*, 2 *B. minimus* groups (north and south of Bay of Biscay) were found. It is probable that this pattern derives from *D. labrax*, this parasite's final host, when specific genomic areas under selection are investigated. However, this structure may also be the result of sampling limitations mostly in the southern beds. The full parasite population connectivity will only be revealed by additional research, specifically by increasing the number of samples of understudied beds, such as in the Mediterranean.

## Supporting information

Correia et al. supplementary material 1 Correia et al. supplementary material

Correia et al. supplementary material 2 Correia et al. supplementary material

## Data Availability

The data that support the findings of this manuscript are provided in the text and are also available from the corresponding authors (S.C. and M.V.) upon reasonable request.

## Supplementary material

The supplementary material for this article can be found at https://doi.org/10.1017/S0031182023000987

## References

[ref1] Abdul-Muneer PM (2014) Application of microsatellite markers in conservation genetics and fisheries management: recent advances in population structure analysis and conservation strategies. Genetics Research International 2014, 691759. doi: 10.1155/2014/691759 24808959 PMC3997932

[ref2] Agola LE, Steinauer ML, Mburu DN, Mungai BN, Mwangi IN, Magoma GN, Loker ES and Mkoji GM (2009) Genetic diversity and population structure of *Schistosoma mansoni* within human infrapopulations in Mwea, central Kenya assessed by microsatellite markers. Acta Tropica 111, 219–225. 19427295 10.1016/j.actatropica.2009.04.012 PMC2763435

[ref3] Alizon S and van Baalen M (2008) Multiple infections, immune dynamics, and the evolution of virulence. American Naturalist 172, E150–E168. 10.1086/590958 18702601

[ref4] AQUATRACE (2017) The development of tools for tracing and evaluating the genetic impact of fish from aquaculture: ‘AquaTrace’. CORDIS EU research accessed 20 March 2023. Available at https://cordis.europa.eu/project/id/311920/reporting

[ref5] Baiocchi T, Lee G, Choe DH and Dillman AR (2017) Host seeking parasitic nematodes use specific odors to assess host resources. Scientific Reports 7, 6270. 28740104 10.1038/s41598-017-06620-2 PMC5524962

[ref6] Balmer O, Stearns SC, Schotzau A and Brun R (2009) Intraspecific competition between co-infecting parasite strains enhances host survival in African trypanosomes. Ecology 90, 3367–3378. 20120806 10.1890/08-2291.1

[ref7] Bartoli P and Gibson DI (2007) Synopsis of the life cycles of Digenea (Platyhelminthes) from lagoons of the northern coast of the western Mediterranean. Journal of Natural History 41, 1553–1570.

[ref8] Bell AS, De Roode JC, Sim D and Read AF (2006) Within-host competition in genetically diverse malaria infections: parasite virulence and competitive success. Evolution 60, 1358–1371. 16929653

[ref9] Bowers EA (1969) Cercariae *Bucephalosis haimeana* (Lacaze-Duthiers, 1854) (Digenea – Bucephalidae) in cockle, *Cardium edule* L. in south Wales. Journal of Natural History 3, 409–422.

[ref10] Burman M (1982) *Neoaplectana carpocapsae*: toxin production by axenic insect parasitic nematodes. Nematologica 28, 62–70.

[ref11] Carballal MJ, Iglesias D, Santamarina J, Ferro-Soto B and Villalba A (2001) Parasites and pathologic conditions of the cockle *Cerastoderma edule* populations of the coast of Galicia (NW Spain). Journal of Invertebrate Pathology 78, 87–97. 11812111 10.1006/jipa.2001.5049

[ref12] Carlson CJ, Dallas TA, Alexander LW, Phelan AL and Phillips AJ (2020) What would it take to describe the global diversity of parasites? Proceedings of the Royal Society B-Biological Sciences 287, 20201841. doi: 10.1098/rspb.2020.1841 PMC7739500 33203333

[ref13] Correia S, Magalhães L, Freitas R, Bazairi H, Gam M and de Montaudouin X (2020) Large scale patterns of trematode parasite communities infecting *Cerastoderma edule* along the Atlantic coast from Portugal to Morocco. Estuarine Coastal and Shelf Science 233, 106546. doi: 10.1016/j.ecss.2019.106546

[ref14] Cribb TH, Bray RA, Olson PD and Littlewood DTJ (2003) Life cycle evolution in the Digenea: a new perspective from phylogeny. Advances in Parasitology 54, 197–254. 14711086 10.1016/s0065-308x(03)54004-0

[ref15] Criscione CD and Blouin MS (2006) Minimal selfing, few clones, and no among-host genetic structure in a hermaphroditic parasite with asexual larval propagation. Evolution 60, 553–562. 16637500

[ref16] Curtis LA, Kinley JL and Tanner NL (2000) Longevity of oversized individuals: growth, parasitism, and history in an estuarine snail population. Journal of the Marine Biological Association of the United Kingdom 80, 811–820.

[ref17] Davies CM, Fairbrother E and Webster JP (2002) Mixed strain schistosome infections of snails and the evolution of parasite virulence. Parasitology 124, 31–38. 11811801 10.1017/s0031182001008873

[ref18] de Montaudouin X, Thieltges DW, Gam M, Krakau M, Pina S, Bazairi H, Dabouineau L, Russell-Pinto F and Jensen KT (2009) Digenean trematode species in the cockle *Cerastoderma edule*: identification key and distribution along the north-eastern Atlantic shoreline. Journal of the Marine Biological Association of the United Kingdom 89, 543–556.

[ref19] de Montaudouin X, Blanchet H, Desclaux-Marchand C, Lavesque N and Bachelet G (2016) Cockle infection by *Himasthla quissetensis* – I. From cercariae emergence to metacercariae infection. Journal of Sea Research 113, 99–107.

[ref20] de Montaudouin X, Arzul I, Cao A, Carballal MJ, Chollet B, Correia S, Cuesta J, Culloty S, Daffe G, Darriba S, Díaz S, Engelsma M, Freitas R, Garcia C, Goedknegt A, Gonzalez P, Grade A, Groves E, Iglesias D, Jensen KT, Joaquim S, Lynch S, Magalhães L, Mahony K, Maia F, Malham S, Matias D, Nowaczyk A, Ruano F, Thieltges D and Villalba A (2021) Parasites and Diseases of the Common Cockle Cerastoderma edule, 1st Edn. Aveiro: UA Editora-Universidade de Aveiro.

[ref21] de Pontual H, Heerah K, Goossens J, Garren F, Martin S, Le Ru L, Le Roy D and Woillez M (2023) Seasonal migration, site fidelity, and population structure of European seabass (*Dicentrarchus labrax*). ICES Journal of Marine Science 80, 1606–1618.

[ref22] Desclaux C, de Montaudouin X and Bachelet G (2002) Cockle emergence at the sediment surface: ‘favourization’ mechanism by digenean parasites? Diseases of Aquatic Organisms 52, 137–149. 12542091 10.3354/dao052137

[ref23] Dubois SY, Savoye N, Sauriau PG, Billy I, Martinez P and de Montaudouin X (2009) Digenean trematodes-marine mollusc relationships: a stable isotope study. Diseases of Aquatic Organisms 84, 65–77. 19419008 10.3354/dao2022

[ref24] Dumont M, Mone H, Mouahid G, Idris MA, Shaban M and Boissier J (2007) Influence of pattern of exposure, parasite genetic diversity and sex on the degree of protection against reinfection with *Schistosoma mansoni*. Parasitology Research 101, 247–252. 17310396 10.1007/s00436-007-0476-0

[ref25] Excoffier L, Laval G and Schneider S (2005) Arlequin (version 3.0): an integrated software package for population genetics data analysis. Evolutionary Bioinformatics Online 1, 47–50. PMC2658868 19325852

[ref26] Feis ME, Thieltges DW, Olsen JL, de Montaudouin X, Jensen KT, Bazairi H, Culloty SC and Luttikhuizen PC (2015) The most vagile host as the main determinant of population connectivity in marine macroparasites. Marine Ecology Progress Series 520, 85–99.

[ref27] Fredensborg BL and Poulin R (2005) Larval helminths in intermediate hosts: does competition early in life determine the fitness of adult parasites? International Journal for Parasitology 35, 1061–1070. 16019005 10.1016/j.ijpara.2005.05.005

[ref28] Fredensborg BL, Mouritsen KN and Poulin R (2005) Impact of trematodes on host survival and population density in the intertidal gastropod *Zeacumantus subcarinatus*. Marine Ecology Progress Series 290, 109–117.

[ref29] Goedknegt MA, Feis ME, Wegner KM, Luttikhuizen PC, Buschbaum C, Camphuysen K, van der Meer J and Thieltges DW (2016) Parasites and marine invasions: ecological and evolutionary perspectives. Journal of Sea Research 113, 11–27.

[ref30] Goujon M, McWilliam H, Li W, Valentin F, Squizzato S, Paern J and Lopez R (2010) A new bioinformatics analysis tools framework at EMBL-EBI. Nucleic Acids Research 38, W695–W699. 20439314 10.1093/nar/gkq313 PMC2896090

[ref31] Hall TA (1999) BioEdit: a user-friendly biological sequence alignment editor and analysis program for Windows 95/98/NT. Nucleic Acids Symposium Series 41, 95–98.

[ref32] Hatcher MJ and Dunn AM (2011) Parasites in Ecological Communities: From Interactions to Ecosystems. Cambridge, UK: Cambridge University Press.

[ref33] Intecmar (2021) Informe Epidemiolóxico de moluscos bivalvos de Galicia, ano 2021. Intecmar – Instituto Tecnoloxico para o controlo do medio marino de Galicia, Unidade de Patoloxía, Galicia, Spain. Available at http://www.intecmar.gal/Informacion/Patoloxia/Default.aspx

[ref34] Johnson PTJ, Dobson A, Lafferty KD, Marcogliese DJ, Memmott J, Orlofske SA, Poulin R and Thieltges DW (2010) When parasites become prey: ecological and epidemiological significance of eating parasites. Trends in Ecology & Evolution 25, 362–371. 20185202 10.1016/j.tree.2010.01.005

[ref35] Jokela J, Uotila L and Taskinen J (1993) Effect of the castrating trematode parasite *Rhipidocotyle fennica* on energy allocation of fresh-water clam *Anodonta piscinalis*. Functional Ecology 7, 332–338.

[ref36] Keeney DB, Waters JM and Poulin R (2007) Clonal diversity of the marine trematode *Maritrema novaezealandensis* within intermediate hosts: the molecular ecology of parasite life cycles. Molecular Ecology 16, 431–439. 17217355 10.1111/j.1365-294X.2006.03143.x

[ref37] Keeney DB, Boessenkool S, King TM, Leung TLF and Poulin R (2008) Effects of interspecific competition on asexual proliferation and genetic diversity in larval trematode infections of snails. Parasitology 135, 741–747. 18442429 10.1017/S0031182008004435

[ref38] Kumar S, Stecher G, Li M, Knyaz C and Tamura K (2018) MEGA X: molecular evolutionary genetics analysis across computing platforms. Molecular Biology and Evolution 35, 1547–1549. 29722887 10.1093/molbev/msy096 PMC5967553

[ref39] Lagrue C, McEwan J, Poulin R and Keeney DB (2007) Co-occurrences of parasite clones and altered host phenotype in a snail–trematode system. International Journal for Parasitology 37, 1459–1467. 17582419 10.1016/j.ijpara.2007.04.022

[ref40] Lemaire C, Versini JJ and Bonhomme F (2005) Maintenance of genetic differentiation across a transition zone in the sea: discordance between nuclear and cytoplasmic markers. Journal of Evolutionary Biology 18, 70–80. 15669962 10.1111/j.1420-9101.2004.00828.x

[ref41] Librado P and Rozas J (2009) DnaSP v5: a software for comprehensive analysis of DNA polymorphism data. Bioinformatics 25, 1451–1452. 19346325 10.1093/bioinformatics/btp187

[ref42] Loewe L and Hill WG (2010) The population genetics of mutations: good, bad and indifferent. Philosophical Transactions of the Royal Society B-Biological Sciences 365, 1153–1167. 10.1098/rstb.2009.0317 PMC2871823 20308090

[ref43] Louhi KR, Karvonen A, Rellstab C, Louhi R and Jokela J (2013) Prevalence of infection as a predictor of multiple genotype infection frequency in parasites with multiple-host life cycle. Journal of Animal Ecology 82, 191–200. 22985060 10.1111/j.1365-2656.2012.02028.x

[ref44] Magalhães L, Freitas R and de Montaudouin X (2015) Review: *Bucephalus minimus*, a deleterious trematode parasite of cockles *Cerastoderma* spp. Parasitology Research 114, 1263–1278. 25681142 10.1007/s00436-015-4374-6

[ref45] Magalhães L, Freitas R, Dairain A and de Montaudouin X (2017) Can host density attenuate parasitism? Journal of the Marine Biological Association of the United Kingdom 97, 497–505.

[ref46] Magalhães L, Correia S, de Montaudouin X and Freitas R (2018) Spatio-temporal variation of trematode parasites community in *Cerastoderma edule* cockles from Ria de Aveiro (Portugal). Environmental Research 164, 114–123. 29486342 10.1016/j.envres.2018.02.018

[ref47] Magalhães L, Daffe G, Freitas R and de Montaudouin X (2020) *Monorchis parvus* and *Gymnophallus choledochus*: two trematode species infecting cockles as first and second intermediate host. Parasitology 147, 643–658. 32127062 10.1017/S0031182020000402 PMC10317668

[ref48] Martel A and Chia FS (1991) Drifting and dispersal of small bivalves and gastropods with direct development. Journal of Experimental Marine Biology and Ecology 150, 131–147.

[ref49] Mideo N (2009) Parasite adaptations to within-host competition. Trends in Parasitology 25, 261–268. 19409846 10.1016/j.pt.2009.03.001

[ref50] Morley NJ (2012) Thermodynamics of miracidial survival and metabolism. Parasitology 139, 1640–1651. 22814411 10.1017/S0031182012000960

[ref51] Morley NJ and Lewis JW (2017) Thermodynamics of egg production, development and hatching in trematodes. Journal of Helminthology 91, 284–294. 27150072 10.1017/S0022149X16000249

[ref52] Moszczynska A, Locke SA, McLaughlin JD, Marcogliese DJ and Crease TJ (2009) Development of primers for the mitochondrial cytochrome c oxidase I gene in digenetic trematodes (Platyhelminthes) illustrates the challenge of barcoding parasitic helminths. Molecular Ecology Resources 9, 75–82. 21564967 10.1111/j.1755-0998.2009.02634.x

[ref53] Mouritsen KN and Poulin R (2002) Parasitism, community structure and biodiversity in intertidal ecosystems. Parasitology 124, S101–S117. 12396219 10.1017/s0031182002001476

[ref54] Nieberding C and Olivieri I (2007) Parasites: proxies for host genealogy and ecology? Trends in Ecology & Evolution 22, 156–165. 17157954 10.1016/j.tree.2006.11.012

[ref55] Nieberding C, Morand S, Libois R and Michaux JR (2004) A parasite reveals cryptic phylogeographic history of its host. Proceeding of the Royal Society B 271, 2559–2568. 10.1098/rspb.2004.2930 PMC1691906 15615681

[ref56] Paradis E (2010) pegas: an R package for population genetics with an integrated-modular approach. Bioinformatics 26, 419–420. 20080509 10.1093/bioinformatics/btp696

[ref57] Paradis E and Schliep K (2019) ape 5.0: an environment for modern phylogenetics and evolutionary analyses in R. Bioinformatics 35, 526–528. 30016406 10.1093/bioinformatics/bty633

[ref58] Pina S, Barandela T, Santos MJ, Russell-Pinto F and Rodrigues P (2009) Identification and description of *Bucephalus minimus* (Digenea: Bucephalidae) life cycle in Portugal: morphological, histopathological and molecular data. Journal of Parasitology 95, 353–359. 18710298 10.1645/GE-1719.1

[ref59] Poulin R (1999) The functional importance of parasites in animal communities: many roles at many levels? International Journal for Parasitology 29, 903–914. 10480727 10.1016/s0020-7519(99)00045-4

[ref60] Poulin R (2001) Interactions between species and the structure of helminth communities. Parasitology 122, S3–S11. 11442194 10.1017/s0031182000016991

[ref61] Poulin R (2006) Global warming and temperature-mediated increases in cercarial emergence in trematode parasites. Parasitology 132, 143–151. 16393363 10.1017/S0031182005008693

[ref62] Preston DL, Orlofske SA, Lambden JP and Johnson PTJ (2013) Biomass and productivity of trematode parasites in pond ecosystems. Journal of Animal Ecology 82, 509–517. 23488451 10.1111/1365-2656.12030

[ref63] Queiroz N, Humphries NE, Noble LR, Santos AM and Sims DW (2012) Spatial dynamics and expanded vertical niche of blue sharks in oceanographic fronts reveal habitat targets for conservation. PLoS ONE 7, e32374. doi: 10.1371/journal.pone.0032374 22393403 PMC3290575

[ref64] Quéré N, Guinand B, Kuhl H, Reinhardt R, Bonhomme F and Desmarais E (2010) Genomic sequences and genetic differentiation at associated tandem repeat markers in growth hormone, somatolactin and insulin-like growth factor-1 genes of the sea bass, *Dicentrarchus labrax*. Aquatic Living Resources 23, 285–296.

[ref65] Rajakumar S, Bleiss W, Hartmann S, Schierack P, Marko A and Lucius R (2006) Concomitant immunity in a rodent model of filariasis: the infection of *Meriones unguiculatus* with *Acanthocheilonema viteae*. Journal of Parasitology 92, 41–45. 16629313 10.1645/GE-3507.1

[ref66] Rauch G, Kalbe M and Reusch TBH (2005) How a complex life cycle can improve a parasite's sex life. Journal of Evolutionary Biology 18, 1069–1075. 16033580 10.1111/j.1420-9101.2005.00895.x

[ref67] Read AF and Taylor LH (2001) The ecology of genetically diverse infections. Science 292, 1099–1102. 11352063 10.1126/science.1059410

[ref68] Rice P, Longden I and Bleasby A (2000) *EMBOSS*: the European molecular biology open software suite. Trends in Genetics 16, 276–277. 10827456 10.1016/s0168-9525(00)02024-2

[ref69] Sasal S, Durand P, Faliex E and Morand S (2000) Experimental approach to the importance of parasitism in biological conservation. Marine Ecology Progress Series 198, 293–302.

[ref70] Schliep K (2011) phangorn: phylogenetic analysis in R. Bioinformatics 27, 592–593. 21169378 10.1093/bioinformatics/btq706 PMC3035803

[ref71] SchulteOehlmann U, Oehlmann J, Fioroni P and Bauer B (1997) Imposex and reproductive failure in *Hydrobia ulvae* (Gastropoda: Prosobranchia). Marine Biology 128, 257–266.

[ref72] Selbach C and Poulin R (2020) Some like it hotter: trematode transmission under changing temperature conditions. Oecologia 194, 745–755. 33170408 10.1007/s00442-020-04800-y

[ref73] Selva L, Viana D, Regev-Yochay G, Trzcinski K, Corpa JM, Lasa I, Novick RP and Penades JR (2009) Killing niche competitors by remote-control bacteriophage induction. Proceedings of the National Academy of Sciences of the USA 106, 1234–1238. 19141630 10.1073/pnas.0809600106 PMC2633583

[ref74] Souche EL, Hellemans B, Babbucci M, MacAoidh E, Guinard B, Bargelloni L, Chistiakov DA, Patarnello T, Bonhomme F, Martinsohn JT and Volckaert FAM (2015) Range-wide population structure of European sea bass *Dicentrarchus labrax*. Biological Journal of the Linnean Society 116, 86–105.

[ref75] Theron A, Sire C, Rognon A, Prugnolle F and Durand P (2004) Molecular ecology of *Schistosoma mansoni* transmission inferred from the genetic composition of larval and adult infrapopulations within intermediate and definitive hosts. Parasitology 129, 57–585. 10.1017/s0031182004005943 15552402

[ref76] Thieltges DW (2004) Effects of trematode parasites on mortality in the common cockle *Cerastoderma edule*. International Journal of Medical Microbiology 293, 57–57.

[ref77] Thieltges DW (2006) Parasite induced summer mortality in the cockle *Cerastoderma edule* by the trematode *Gymnophallus choledochus*. Hydrobiologia 559, 455–461.

[ref78] Thieltges DW and Rick J (2006) Effect of temperature on emergence, survival and infectivity of cercariae of the marine trematode *Renicola roscovita* (Digenea: Renicolidae). Diseases of Aquatic Organisms 73, 63–68. 17240753 10.3354/dao073063

[ref79] Vandeputte M, Gagnaire PA and Allal F (2019) The European sea bass: a key marine fish model in the wild and in aquaculture. Animal Genetics 50, 195–206. 30883830 10.1111/age.12779 PMC6593706

[ref80] Vera M, Maroso F, Wilmes SB, Hermida M, Blanco A, Fernández C, Groves E, Malham SK, Bouza C, Cockle's Consortium, Robins PE and Martínez P (2022) Genomic survey of edible cockle (*Cerastoderma edule*) in the Northeast Atlantic: a baseline for sustainable management of its wild resources. Evolutionary Applications 15, 262–285. 35233247 10.1111/eva.13340 PMC8867702

[ref81] Vilas R, Paniagua E and Sanmartin ML (2003) Genetic variation within and among infrapopulations of the marine digenetic trematode *Lecithochirium fusiforme*. Parasitology 126, 465–472. 12793651 10.1017/s0031182003003081

[ref82] Yu G, Smith D, Zhu H, Guan Y and Lam TT (2017) ggtree: and R package for visualization and annotation of phylogenetic trees with their covariates and other associated data. Methods in Ecology and Evolution 8, 28–36.

[ref83] Zemmer SA, Detwiler JT, Sokol ER, Da Silva Neto JG, Wyderko J, Potts K, Gajewski ZJ, Sarment LV, Benfield EF and Belden LK (2020) Spatial scale and structure of complex life cycle trematode parasite communities in streams. PLOS ONE 15, e0241973. doi: 10.1371/journal.pone.0241973 33232346 PMC7685432

