# Supplementary material for: Trematode genetic patterns at host individual and population scales provide insights about infection mechanisms
Source: Parasitology. 2023 Oct 20;150(13):1207–20. doi: 10.1017/S0031182023000987 (PMC10941227; doi:10.1017/S0031182023000987)
Supplement: Correia et al. supplementary material 1 — Correia et al. supplementary material [file S0031182023000987sup001.pdf]

Table S1. Pairwise Φ_{ST} values between cockle beds included in the present study. Non-significant values are indicated in bold letters.

	Merja Zerga	Aveiro	Baiona	Noia	Arcachon	Bay of Somme	English Channel	Celtic Sea	Burry Inlet	The Dee	Wadden Sea
Merja Zerga	0.0000										
Aveiro	0.5194	0.0000									
Baiona	0.8442	0.5135	0.0000								
Noia	**0.9082**	**0.0241**	**0.8414**	0.0000							
Arcachon	0.3889	**0.0758**	0.4528	0.4962	0.0000						
Bay of Somme	0.7979	**0.1178**	0.7547	**0.7268**	**-0.0338**	0.0000					
English Channel	0.6253	0.2306	0.7099	0.7444	**0.0232**	**-0.0315**	0.0000				
Celtic Sea	0.4406	0.1521	0.5617	**0.5602**	0.0530	**0.0040**	0.0648	0.0000			
Burry Inlet	0.7376	**-0.1354**	0.7612	**0.8257**	**-0.2122**	**-0.0964**	**-0.1331**	**-0.2930**	0.0000		
The Dee	0.8437	**0.0675**	0.7596	**0.6173**	**-0.0050**	**-0.0453**	**0.1042**	**-0.0631**	**0.0004**	0.0000	
Wadden Sea	0.6333	0.2305	0.7175	0.7397	0.0622	**-0.0042**	**-0.0004**	0.0664	**-0.1340**	**0.0665**	0.0000

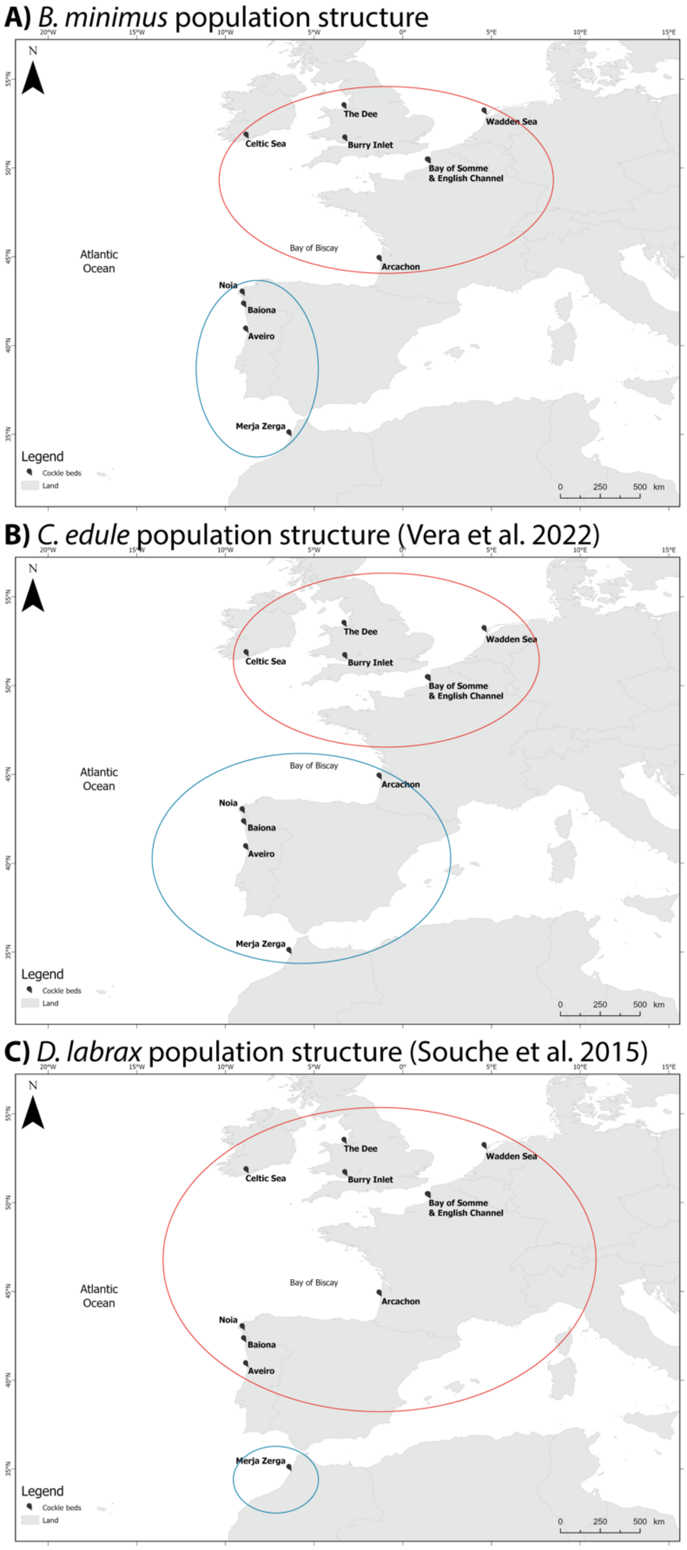

Figure S1. Contrasting population genetic structure of **A)** *Bucephalus minimus* (found in this study), **B)** *B. minimus* first intermediate host, *Cerastoderma edule* (adapted from Vera et al. 2022), and **C)** *B. minimus* definitive host, *Dicentrarchus labrax* (adapted from Souche et al. 2015).
