# Supplementary figures and images for: Trematode genetic patterns at host individual and population scales provide insights about infection mechanisms
Source: Parasitology. 2023 Oct 20;150(13):1207–20. doi: 10.1017/S0031182023000987 (PMC10941227; doi:10.1017/S0031182023000987)

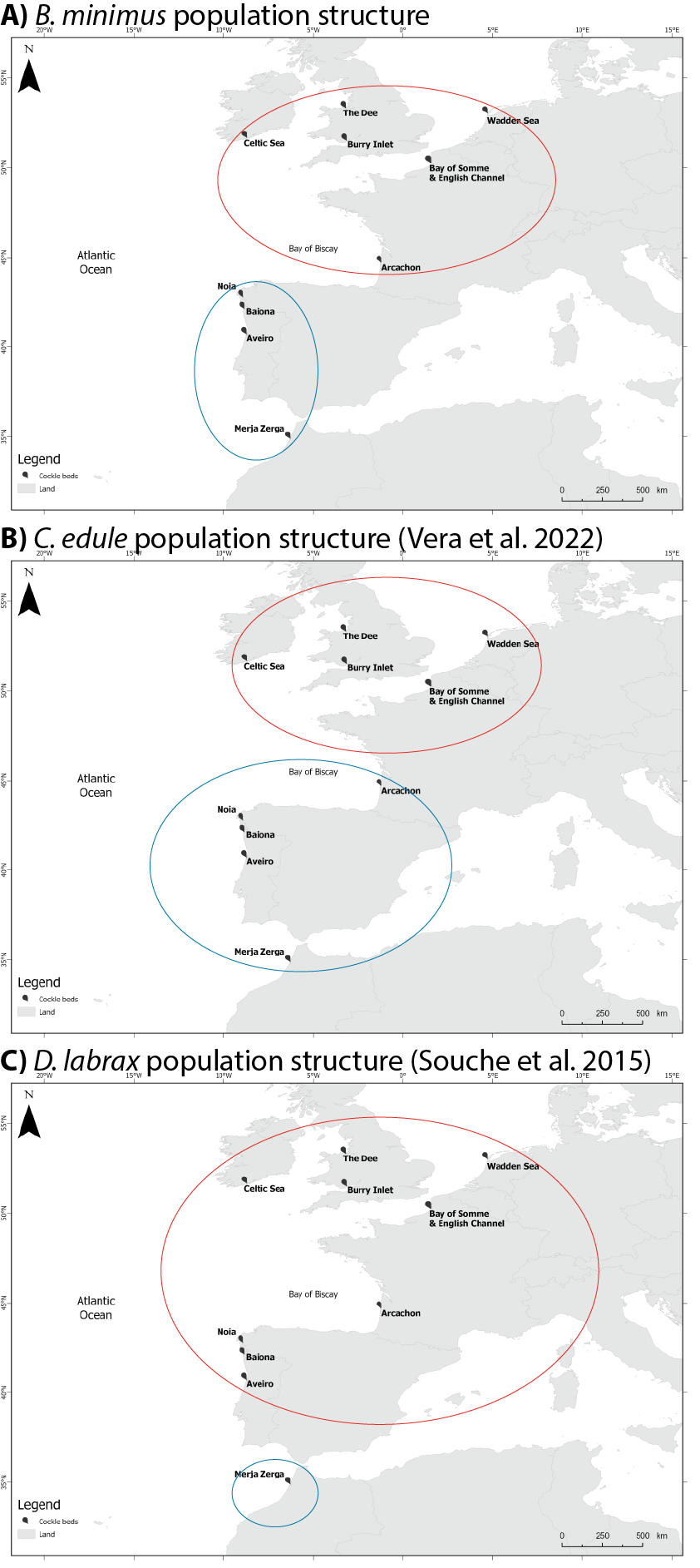

Supplement: Correia et al. supplementary material 2 — Correia et al. supplementary material [file S0031182023000987sup002.tif]
